# Plasma proteoforms of apolipoproteins C-I and C-II are associated with plasma lipids in the Multi-Ethnic Study of Atherosclerosis

**DOI:** 10.1016/j.jlr.2022.100263

**Published:** 2022-08-09

**Authors:** Juraj Koska, Jeremy Furtado, Yueming Hu, Shripad Sinari, Matthew J. Budoff, Dean Billheimer, Dobrin Nedelkov, Robyn L. McClelland, Peter D. Reaven

**Affiliations:** 1 Phoenix VA Health Care System, Phoenix, AZ, USA; 2 Department of Nutrition, Harvard T.H. Chan School of Public Health, Boston, MA, USA; 3 Isoformix Inc, Phoenix, AZ, USA; 4 Mel and Enid Zuckerman College of Public Health, University of Arizona, Tucson, AZ, USA; 5 Los Angeles Biomedical Research Institute, Torrance, CA, USA; 6 Department of Biostatistics, University of Washington, Seattle, WA, USA; 7 College of Health Solutions, Arizona State University, Phoenix, AZ, USA

**Keywords:** apolipoprotein posttranslational proteoforms, atherosclerosis, cholesterol, HDL, lipid metabolism, lipid transport, mass spectrometry, proteomics, race/ethnicity, triglycerides, apoC-I, apolipoprotein C-I, apoC-II, apolipoprotein C-II, DPP-4, dipeptidyl peptidase-4, GFR, glomerular filtration rate, LPL, lipoprotein lipase, MESA, Multi-Ethnic Study of Atherosclerosis, TRL, triglyceride-rich lipoprotein

## Abstract

Apolipoproteins (apo) C-I and C-II are key regulators of triglyceride and HDL metabolism. Both exist as full-size native and truncated (apoC-I'; apoC-II') posttranslational proteoforms. However, the determinants and the role of these proteoforms in lipid metabolism are unknown. Here, we measured apoC-I and apoC-II proteoforms by mass spectrometry immunoassay in baseline and 10-year follow-up plasma samples from the Multi-Ethnic Study of Atherosclerosis. We found that baseline total apoC-I (mean = 9.2 mg/dl) was lower in African Americans (AA), Chinese Americans (CA), and Hispanics (by 1.8; 1.0; 1.0 mg/dl vs. whites), higher in women (by 1.2 mg/dl), and positively associated with plasma triglycerides and HDL. Furthermore, we observed that the truncated-to-native apoC-I ratio (apoC-I'/C-I) was lower in CA, negatively associated with triglycerides, and positively associated with HDL. We determined that total apoC-II (8.8 mg/dl) was lower in AA (by 0.8 mg/dl) and higher in CA and Hispanics (by 0.5 and 0.4 mg/dl), positively associated with triglycerides, and negatively associated with HDL. In addition, apoC-II'/C-II was higher in AA and women, negatively associated with triglycerides, and positively associated with HDL. We showed that the change in triglycerides was positively associated with changes in total apoC-I and apoC-II and negatively associated with changes in apoC-I'/C-I and apoC-II'/C-II, whereas the change in HDL was positively associated with changes in total apoC-I and apoC-II'/C-II and negatively associated with change in total apoC-II. This study documents racial/ethnic variation in apoC-I and apoC-II plasma levels and highlights apolipoprotein posttranslational modification as a potential regulator of plasma lipids.

Apolipoproteins C-I and C-II are small exchangeable molecules produced mainly by liver and present on several classes of lipoprotein particles where they play an important role in regulating lipid metabolism (1, 2). Apolipoprotein C-I (apoC-I) is a major protein moiety of triglyceride-rich lipoproteins (TRLs), including chylomicrons and VLDL, and HDL, and to a lesser extent is present in LDL (1, 3). ApoC-I participates in lipid transport and metabolism and rapidly exchanges between lipoprotein classes. It has effects both on lipoprotein receptors by inhibiting binding mediated by apolipoprotein E and modulation of activities of several enzymes (1, 3, 4). For example, apoC-I has been associated with inhibition of lipoprotein lipase (LPL), hepatic lipase, phospholipase A2, and cholesteryl ester transfer protein and activation of LCAT. Apolipoprotein C-II (apoC-II) exchanges between HDL in the fasting state and TRL postprandially (2, 3, 4). ApoC-II also plays a critical role in TRL metabolism, primarily by acting as activating cofactor of LPL (5, 6). ApoC-II deficiency results in type I hyperlipoproteinemia and has been linked with atherosclerosis (2, 7, 8).

Upon intracellular cleavage of 20 and 22 amino acid signal peptides, apoC-I and apoC-II are released into circulation as full-size native proteins containing 57 and 79 amino acids, respectively. In the circulation, both undergo further truncation and appear as native proteoforms along with lesser concentrations of truncated proteoforms (Fig. 1A, B) (5, 9, 10, 11). The enzyme responsible for removing the two N-terminal amino acids of apoC-I is dipeptidyl peptidase-4 (DPP-4) (12), a widely expressed enzyme cleaving many peptides, including chemokines, neuropeptides, and hormones (13). DPP-4 inhibitors block cleavage of incretin hormones glucagon-like peptide 1 and glucose-dependent insulinotropic polypeptide (13), modify glucose and postprandial lipid levels, and are an approved medication for type 2 diabetes. The removal of the six N-terminal amino acids from apoC-II yields a proteoform termed mature apoC-II (apoC-IIʹ). We and others have recently demonstrated that posttranslational modifications of native apoC-III vary substantially among individuals and have unique relationships with lipid levels (14, 15). In contrast, the determinants of apoC-I and C-II proteoforms in plasma and their associations with lipid levels in vivo are completely unknown.

**Fig. 1:**
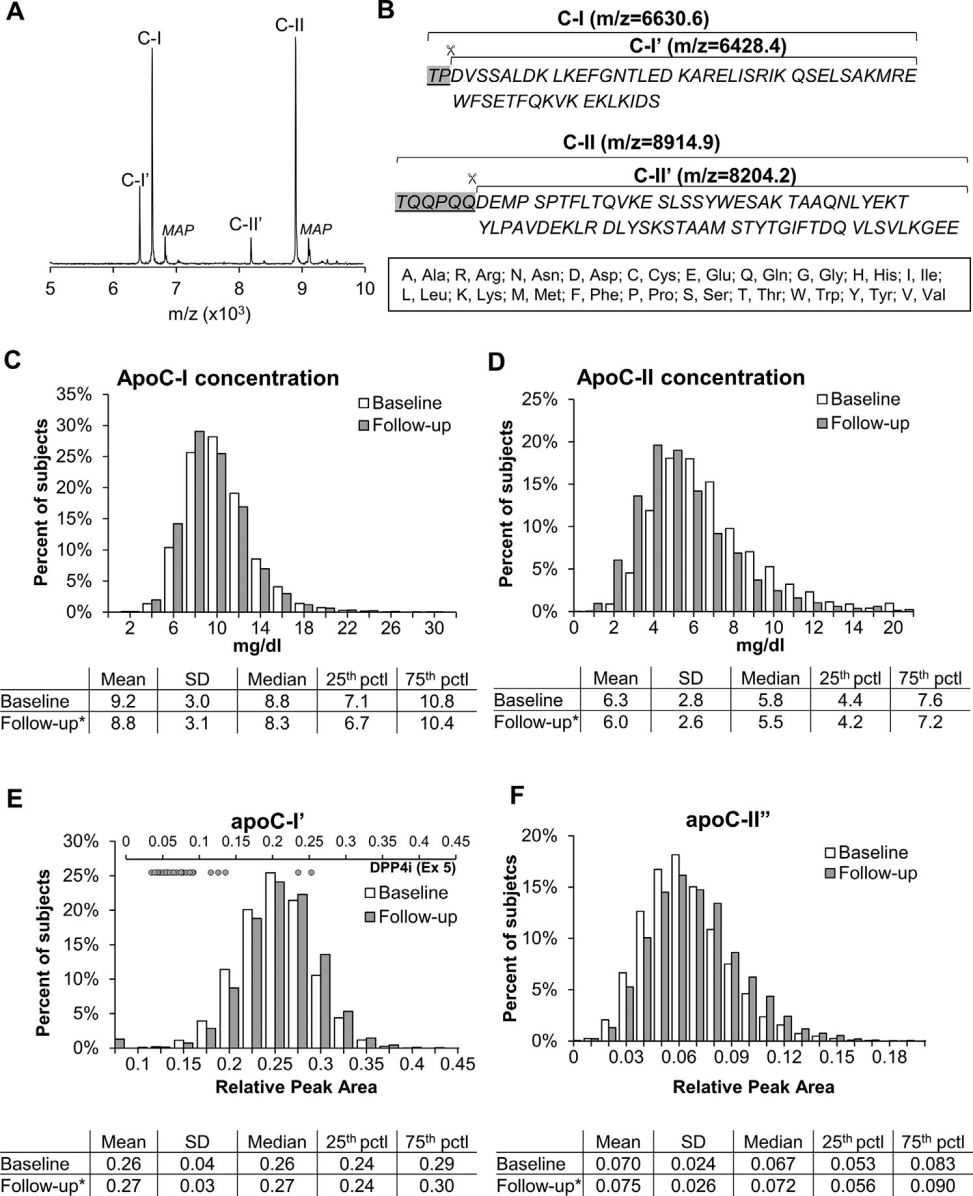
Representative mass spectra (A) and amino acid sequences (B) of native and truncated (indicated by an apostrophe) apoC-I and apoC-II proteoforms. MAP, MALDI matrix adduct peaks (+220 Da of native apoC-I and apoC-II). C–F: Distribution of apoC-I and apoC-II plasma total concentrations and relative peak areas of truncated proteoforms at baseline and follow-up. Embedded tables indicate descriptive statistics for each apoC-I and apoC-II measure. ∗*P* < 0.05 for differences between baseline and follow-up. Inset figure on top of panel E shows individual levels of truncated apoC-I in participants with reported use of dipeptidyl-peptidase 4 inhibitors (DPP-4is) at follow-up.

In the current study, we measured both absolute concentrations of apoC-I and apoC-II as well as relative amounts of their proteoforms in the Multi-Ethnic Study of Atherosclerosis (MESA). MESA is a large community-based cohort that has collected baseline and follow-up data on participant characteristics, health status, medication use, and plasma lipids. We examined the cross-sectional correlates of total and truncated apoC-I and apoC-II measures and determined cross-sectional and longitudinal relationships of these measures with plasma lipids.

## Materials and methods

### Study population

The MESA study is a multicenter longitudinal study to examine factors associated with subclinical CVD and the progression from subclinical to clinical CVD in individuals aged 45–84 years and without known CVD at baseline (16). The current study used plasma samples collected at exam 1, between July 2000 and August 2002, and at exam 5, approximately 10 years later. For MESA, and the current study, exam 1 data were considered baseline data. The final sample set for this study included 5,791 samples for baseline and 3,851 samples from participants returning for “follow-up” at exam 5. The study was conducted according to the regulations established by the Declaration of Helsinki. Informed consent was obtained from all study participants and institutional review boards at each MESA study site (Columbia University, New York, NY; Johns Hopkins University, Baltimore, MD; Northwestern University, Chicago, IL; University of California at Los Angeles, Los Angeles, CA; University of Minnesota, St. Paul, MN; and Wake Forest University, Winston-Salem, NC) approved the study protocol. Present study was approved by the Phoenix VA Health Care System Institutional Review Board. Demographic information, medical history, and physical measures were obtained through standardized protocols as described previously (17). Blood biomarkers were measured from fasting plasma samples at the MESA central laboratory at the University of Minnesota.

### Mass spectrometry immunoassay

ApoC-I and apoC-II proteoform distribution was measured by mass spectrometry immunoassay as described previously (10) and detailed in supplemental data. Briefly, after thawing on ice, plasma samples were diluted 160-fold in PBS containing 0.1% Tween-20. Using immunoaffinity columns derivatized with anti-apoC-I and anti-apoC-II antibodies (Academy Biomedical Co, Houston, TX), apoC-I and apoC-II proteins were captured from the analytical samples during repeated aspiration and dispensing cycles. Captured proteins were then eluted directly onto a 96-well formatted MALDI target using a sinapinic acid matrix solution. Linear mass spectra were acquired from each sample spot using a Bruker's Ultraflex III MALDI-TOF instrument (Bruker, Billerica, MA) in positive ion mode. Mass spectra were internally calibrated and further processed with Flex Analysis 3.0 software (Bruker Daltonics). All peaks representing apoC-I and C-II were integrated baseline-to-baseline using Zebra 1.0 software (Intrinsic Bioprobes, Inc). Representative mass spectra and proteoform amino acid sequences are shown in Fig. 1A, B. To obtain the percent abundance of truncated apoC-I proteoform (apoC-I'), the peak area of apoC-I' was divided by the added peak areas of both apoC-I' and native apoC-I. The percent abundance of truncated apoC-II proteoform (apoC-II') was similarly calculated. Mean intra-assay coefficients of variations were 4.6% and 1.5% for apoC-I' and native apoC-I and 7.3% and 0.5% for apoC-II' and native apoC-II, respectively. Mean between-assay coefficients of variations were 5.5% and 1.8% for apoC-I' and native apoC-I and 9.1% and 0.6% for apoC-II' and native apoC-II, respectively.

### Biochemical assays

Plasma concentrations of total apoC-I and apoC-II were determined in 3,851 participants who had available samples from both examinations as a part of an ongoing substudy testing the effect of apoC-I and apoC-II measures on measures of cognitive function (available at exam 5). The analyses were performed in duplicates using sandwich ELISAs with identical detection antibodies as used in the proteoform assays above, as described previously (18) and modified herein for measurement of apoC-I and apoC-II in plasma (supplement Methods). Mean intra-assay coefficients of variations were 6% for apoC-I and 4% for apoC-II. Mean between-assay coefficients of variations were 7% for both apoC-I and apoC-II. The concentration of each proteoform was obtained by multiplying the total concentrations by the relative peak area obtained by mass spectrometry immunoassay.

Fasting triglycerides were measured in plasma after an 8-h fast using a glycerol-blanked enzymatic method (Trig/GB; Roche Diagnostics, Indianapolis, IN). Plasma HDL cholesterol was measured by the cholesterol oxidase method (Roche Diagnostics) after precipitation of non-HDL-C magnesium/dextran. LDL cholesterol was calculated by the Friedewald equation (19). Serum glucose was measured using the glucose oxidase method on the Vitros analyzer (Johnson & Johnson, Rochester, NY). Serum creatinine for estimation of glomerular filtration rate (GFR) by MDRD equation was measured by rate reflectance spectrophotometry using thin film adaptation of the creatine amidinohydrolase method on the Vitros analyzer.

### Statistical analyses

Statistical analyses were conducted using SAS, version 9.4 (SAS Institute, Cary, NC). *P* values <0.05 were considered statistically significant. Spearman correlations were used to describe the unadjusted associations between total plasma concentration and truncated-to-native proteoform ratios of apoC-I (apoC-I'/C-I) and apoC-II (apoC-II'/C-II). Repeated-measures linear regression was used to examine the changes in apoC measures and plasma lipids between baseline and follow-up. Multivariable linear regression was used to test for independent associations of apoC-I and apoC-II measures with age, gender, race/ethnicity, BMI, fasting serum glucose, use of lipid-lowering medications, and GFR at baseline and to test the association between changes in apoC measures and changes in plasma lipids (follow-up adjusted for baseline and key clinical and demographic characteristics listed above). In sensitivity analyses, we tested the relationships of baseline apoC-I and apoC-II proteoform ratios with clinical and demographic risk factors separately in those with and without measurements of total apoC concentrations. All continuous variables were natural log transformed and standardized to 1 SD to allow direct comparison of effects.

## Results

### Study population

The general characteristics of the cohort at baseline and follow-up are shown in supplemental Table S1. As previously reported, the MESA participants included nearly equal numbers of men and women and were racially and ethnically diverse, and with relatively low rates of diabetes, dyslipidemia, and renal disease at baseline. Although nearly 50% had a history of hypertension, mean blood pressure levels were well within normal ranges. At baseline, 17% of participants were on lipid-lowering medications, which were primarily statins (91% of the time).

### Total apoC-I and apoC-II concentrations and apoC-I and apoC-II proteoforms

Distributions for total plasma concentrations and truncated proteoform amounts at each visit, including descriptive characteristics, are shown in Fig. 1. Total apoC-I and apoC-II concentrations were significantly lower at follow-up compared with baseline (*P* < 0.0001) (Fig. 1C, D). In contrast, there was shift toward higher relative amounts of apoC-I' and apoC-II' between baseline and follow-up (*P* < 0.0001) (Fig. 1E, F). Despite the rise in the relative amount of apoC-I' over time in the whole group, we also noted that 55 participants had unusually low relative amounts (proportion of peak area <0.1) of apoC-I' at follow-up (Fig. 1E, upper axis). Of this group, 28 (51%) reported use of DPP-4 inhibitors, whereas only five participants disclosed use of these agents in the rest of the cohort. The median relative amount for apoC-I' for those using DPP-4 inhibitors was reduced by 72%. Supplemental Table S2 shows correlations between total apoC-I and apoC-II concentrations and their individual proteoform ratios at baseline and follow-up. The associations between total concentrations and proteoform ratios for each apoC were weaker (rho values = 0.11–0.27) than the associations between total apoC-I and apoC-II (0.57, both exams) or between apoC-I and apoC-II proteoform ratios (0.36 baseline; 0.41 follow-up).

### Association of apoC-I and apoC-II measures with clinical and demographic characteristics

The patterns of association with key demographic and clinical characteristics differed substantially between apoC-I and apoC-II and between total apoC concentrations (both apoC-I and apoC-II) and their truncated/native proteoform ratios (Table 1). All apoC measures except for apoC-I'/C-I were negatively associated with age. Compared with non-Hispanic white participants, total apoC-I and apoC-II were lower, and apoC-I'/C-I and apoC-II'/C-II were higher in African Americans; total apoC-I was lower and apoC-I'/C-I was higher in Hispanic participants; and both total apoC-I and apoC-I'/C-I were lower and total apoC-II was higher in Chinese Americans. Total apoC-I, apoC-I'/C-I, and apoC-II'/C-II were negatively associated, and total apoC-II was positively associated with BMI. Total apoC-II was positively and apoC-II'/C-II was negatively associated with fasting glucose levels. Lower total apoC-I and higher total apoC-II and apoC-II'/C-II were associated with lipid-lowering therapy. All apoC-I and apoC-II measures were inversely associated with estimation of GFR.

**Table 1:** Multivariable association of total concentrations and truncated-to-native proteoform ratios of apoC-I and apoC-II with demographic and clinical characteristics at baseline

Variable	Total apoC-I	apoC-I'/C-I	Total apoC-II	apoC-II'/C-II
n	3,851	5,791	3,851	5,791
Age (1 SD)	−0.07 (0.02)^a^	0.01 (0.01)	−0.06 (0.02)^b^	−0.05 (0.01)^b^
Gender (women)	0.43 (0.03)^a^	−0.02 (0.03)	0.06 (0.03)^c^	0.32 (0.03)^a^
Race/ethnicity (vs. NHW)				
African American	−0.63 (0.04)^a^	0.24 (0.03)^a^	−0.36 (0.04)^a^	0.59 (0.03)^a^
Hispanic	−0.32 (0.04)^a^	0.12 (0.03)^b^	0.07 (0.04)	0.06 (0.03)
Chinese American	−0.30 (0.05)^a^	−0.35 (0.04)^a^	0.21 (0.05)^b^	−0.05 (0.04)
BMI (1 SD)	−0.05 (0.02)^c^	−0.21 (0.01)^a^	0.08 (0.02)^a^	−0.10 (0.01)^a^
Fasting glucose (1 SD)	0.01 (0.02)	−0.13 (0.01)^a^	0.15 (0.02)^a^	−0.02 (0.01)
Lipid-lowering therapy	−0.12 (0.04)^d^	−0.01 (0.03)	0.10 (0.04)^c^	0.17 (0.03)^a^
eGFR (1 SD)	−0.09 (0.02)^a^	−0.14 (0.01)^a^	−0.13 (0.02)^a^	−0.09 (0.01)^a^

eGFR, estimated GFR (MDRD equation); NHW, non-Hispanic white.

Data are β-estimates (SE).

All continuous variables were standardized to 1 SD of natural log-transformed values, and categorical variables are compared with the reference group.

a *P* < 0.0001.

b *P* < 0.001.

c P< 0.05.

d *P* < 0.01.

### Cross-sectional and longitudinal association of apoC-I and apoC-II measures with plasma lipids

**Fig. 2:**
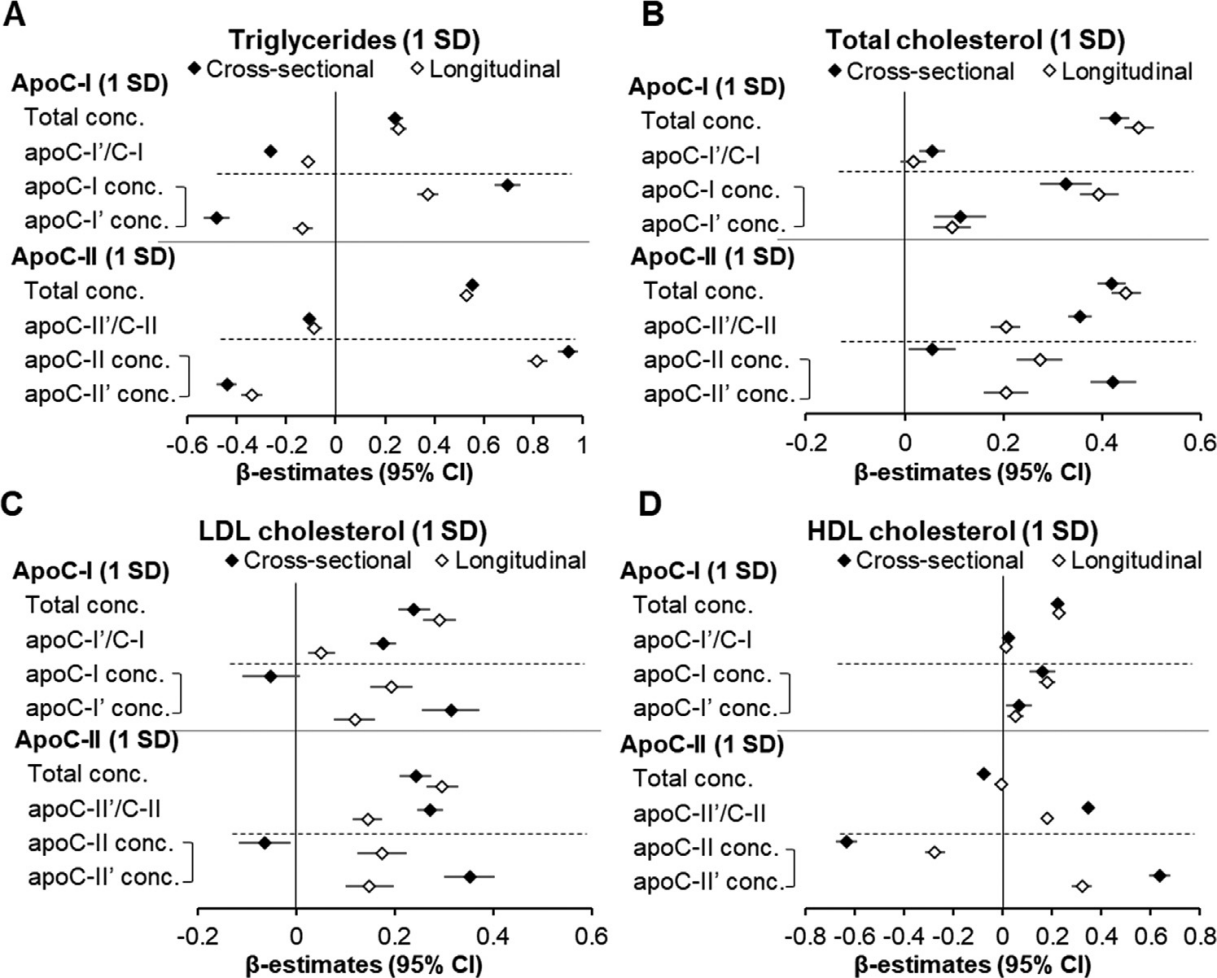
Cross-sectional (baseline) and longitudinal (follow-up adjusted for baseline) relationships of plasma lipids with total plasma concentrations, truncated-to-native proteoform ratios, and individual proteoform concentrations of apoC-I and apoC-II. Concentrations of native and truncated proteoforms for each apoC type were tested in the same model, represented by brackets. All models were adjusted for baseline age, gender, race/ethnicity, BMI, fasting glucose, use of lipid-lowering therapy, and estimated GFR. Longitudinal models also included BMI, fasting glucose, use of lipid-lowering therapy, and estimated GFR at follow-up. All apoC measures were standardized to 1 SD of natural log-transformed values. Sample sizes: n = 5,791 for cross-sectional apoC-I'/C-I and apoC-II'/C-II; n = 3,851 for cross-sectional total and individual proteoform concentrations and for all longitudinal analyses.

Between baseline and follow-up (median = 9.4 years), there was a reduction in plasma triglycerides (median = −12 mg/dl), total cholesterol (−7 mg/dl), and LDL cholesterol (−9 mg/dl), and an increase in plasma HDL cholesterol (4 mg/dl) (all *P* < 0.0001). As demonstrated in Fig. 2, there were distinct patterns of relationships between total concentrations and ratios of apoC-I and apoC-II proteoforms and major lipid species, both in *cross-sectional* assessments at baseline and *longitudinally through follow-up* (by testing associations between *follow-up adjusted for baseline* apoC measures and plasma lipids). Plasma triglycerides were positively associated with total apoC-I and apoC-II concentrations both cross-sectionally and longitudinally (Fig. 2A). In contrast, there were negative associations between apoC-I'/C-I and apoC-II'/C-II and plasma triglycerides in both cross-sectional and longitudinal analyses. When concentrations of native and truncated proteoforms for each individual apoC (calculated from total concentrations and relative amounts) were included in the same models, higher native and lower truncated apoC-I and C-II proteoform concentrations were associated with higher triglycerides. The associations of total or LDL cholesterol with total apoC-I and apoC-II or their proteoform measures were mostly positive, except for an inverse association between native apoC-I and apoC-II and LDL cholesterol at baseline (Fig. 2B, C). Plasma HDL cholesterol was positively associated with total, native, and truncated apoC-I concentrations (Fig. 2D). There was no association between apoC-I'/C-I and HDL. In contrast, HDL was negatively associated with total and native apoC-II concentrations and positively associated with apoC-II'/C-II and apoC-II' concentrations, both sectionally and longitudinally.

As gender and race/ethnicity appeared more closely linked with differences in total apoC-I and apoC-II and/or their proteoform ratios than other participant characteristics, these relationships were examined in the context of triglycerides and HDL levels. In multivariable models, the lower plasma triglycerides present in African Americans (standardized β-estimate = −0.53 [95% CI: −0.59, −0.47] before accounting for apoC measures) were in part attenuated after adjustment for total apoC-I or apoC-II concentrations (−0.39 [−0.47, −0.31], i.e., 26% attenuation; −0.33 [−0.40, −0.27], i.e., 30% attenuation, respectively), whereas sex differences in HDL cholesterol (standardized β-estimate = 0.78 [0.74, 0.83] women vs. men) were attenuated after adjustment for apoC-II'/C-II (0.67 [0.63, 0.71], i.e., 14% attenuation).

The relationships of apoC-I'/C-I and apoC-II'/C-II with demographic and clinical variables including plasma lipids was similar between those with and without total apoC-I and apoC-II measurements (supplemental Table S3).

## Discussion

The present study identified several novel relationships of apoC-I and apoC-II proteoform distribution as well as relationships of total plasma concentrations of both apolipoproteins with key demographic and clinical characteristics and with cross-sectional and longitudinal patterns of plasma lipids. As summarized in Table 2, relative amounts of truncated apoC-I and apoC-II proteoforms frequently had very different relationships with age, gender, race/ethnicity, BMI, lipid-lowering therapy, and fasting plasma glucose, triglycerides, and HDL cholesterol levels than did total concentrations of these apolipoproteins.

**Table 2:** Summary of key relationships of apoC-I and apoC-II total concentrations and truncated-to-native proteoform ratios (C-I'/C-I and C-II'/C-II) with clinical and demographics characteristics and plasma lipids

Characteristic	Total apoC-I	apoC-I'/C-I	Total apoC-II	apoC-II'/C-II
Age	↓	↔	↓	↓
Women	↑	↔	↑	↑
Race/ethnicity (vs. non-Hispanic white)				
African American	↓	↑	↓	↑
Hispanic American	↓	↑	↔	↔
Chinese American	↓	↓	↑	↔
BMI	↓	↓	↑	↓
Fasting glucose	↔	↓	↑	↔
Lipid-lowering therapy	↓	↔	↑	↑
Triglycerides				
Cross-sectional	↑	↓	↑	↓
Longitudinal	↑	↓	↑	↓
HDL cholesterol				
Cross-sectional	↑	↔	↓	↑
Longitudinal	↑	↔	↔	↑

Associations: Cross-sectional—between exam 1 apoC measures and lipids; longitudinal—follow-up adjusted for baseline for both apoC measures and lipids. ↑, positive association; ↓, negative association; ↔, no association.

One key outcome of this study is a comprehensive characterization of concentrations and distribution of apoC-I and apoC-II and their proteoforms in plasma from a large multiethnic cohort, thereby providing valuable reference ranges for these measures. Importantly, our study provides for the first time, the measurement of apoC-I and apoC-II proteoforms in parallel with the total plasma concentrations. Consistent with previous small studies in humans using electro gel techniques for apoC-II (11) or mass spectrometry for apoC-I and apoC-II (9), we demonstrated the presence of a native form (full length, the major form in plasma) and a minor truncated form for both apolipoproteins. The relationships between total concentrations of both apolipoproteins and their proteoform ratios were relatively weak, indicating potential independence of their formation and/or clearance pathways on total apoC-I and apoC-II levels. Consistent with reports that DPP-4 is the enzyme catalyzing apoC-I truncation (20), the relative amounts of apoC-I' at follow-up (when DPP-4 inhibitors were available) were substantially lower in the majority of participants who were receiving DPP-4 inhibitors.

All apoC measures except for apoC-I'/C-I were negatively associated with age and further declined between baseline and follow-up. The significant inverse associations of age with total apoC-I and apoC-II confirm the trends shown for each of these individually in previous smaller studies (21, 22). Other important determinants of apoC-I or apoC-II levels were sex and race/ethnicity. Similar to a previous report (23), total apoC-I concentrations were substantially higher in female participants. We show for the first time that women have also a slightly higher total apoC-II and a more substantial elevation in apoC-II'/C-II. Most prominent among the racial differences in apoC-I and apoC-II measures, African Americans had substantially lower total apoC-I and apoC-II and higher truncated-to-native ratios of both compounds. Importantly, apoC-I and apoC-II measures may in part explain known racial and gender differences in plasma triglycerides and HDL (24, 25). Lower triglycerides in African Americans were partly accounted for by lower total apoC-I and apoC-II, whereas higher HDL-cholesterol in women was partially accounted for by their higher apoC-II'/C-II.

ApoC-I appears to regulate several enzymes involved in triglyceride clearance and cholesterol exchange. Via inhibition of LPL (26, 27, 28), apoC-I may prolong clearance of TRL. In support of this, transgenic mice overexpressing human apoC-I have severe hypertriglyceridemia (26). Consistent with a previous smaller study in humans showing positive correlations of plasma triglycerides with total plasma or VLDL apoC-I levels (21), we found strong positive associations between baseline triglycerides and total apoC-I and between 10-year increases in total apoC-I and triglycerides. In contrast, higher apoC-I'/C-I ratio was associated with lower triglycerides, indicating that native apoC-I may be the proteoform responsible for LPL inhibition and reduced triglyceride clearance.

In the fasting state, up to 70% of apoC-I is present on HDL (29). In vitro evidence suggests a role for apoC-I in the exchange of esterified cholesterol between lipoprotein particles and removal of cholesterol from tissues via inhibition of cholesteryl ester transfer protein and activation of LCAT (30, 31). Consistent with this action, our study showed a strong positive cross-sectional and longitudinal association between total apoC-I and HDL cholesterol.

ApoC-II is an activating cofactor for LPL (6). Contrary to this lipolysis-activating effect, our analyses and several previous studies reported positive associations between total apoC-II and triglyceride concentrations (32, 33). Our finding of a strong independent association of total apoC-II concentrations with both BMI and fasting glucose may provide clues to understanding this surprising relationship. Both obesity and elevated glucose, via multiple mechanisms, contribute to increased VLDL production (34, 35). Detailed kinetic studies have shown positive associations between VLDL production and apoC-II levels, whereas VLDL clearance was not associated with apoC-II levels (36). Thus, the positive association between total apoC-II and triglycerides may reflect increased VLDL production in the setting of overweight/obesity and increased glucose.

It has been hypothesized that since LPL activation occurs at relatively low apoC-II concentrations (37), the expected negative associations between apoC-II concentration and triglyceride levels (via increased clearance) might be masked by a relative excess of total apoC-II on VLDL particles (36). However, our study may provide an alternative explanation that LPL activation is primarily driven by apoC-II', which although a minor subfraction of total apoC-II (∼7%), may be sufficient to activate LPL. This is supported by our finding of an inverse association between apoC-II' concentrations and plasma triglycerides in the combined model, which included concentrations of both apoC-II proteoforms. In vivo, apoC-II regulates LPL in tight concert with other apolipoproteins, plasma proteins, and some lipolysis products. Thus, accurate in vitro assessment of LPL activity requires near physiological substrate systems to mimic this complex system (37). Of note, the original in vitro study showing similar activation of LPL by both apoC-II proteoforms tested purified apoC-II proteoforms on a simple artificial substrate (5). More appropriate in vitro and in vivo systems are likely warranted to test whether and how apoC-II proteoforms differ in their regulation of triglyceride metabolism. Unlike the documented role of DPP-4 in apoC-I truncation, the enzyme responsible for apoC-II truncation is unknown. Based on the finding that native apoA-I (pre-apoA-I) also undergoes cleavage of an N-terminal hexapeptide in an area with partial overlapping amino acid sequence (38, 39), it could be speculated that both apoC-II truncation and apoA-I maturation are catalyzed by the same endopeptidase. Thus, increased cholesterol efflux because of increased formation of mature apoA-I may explain the positive association between apoC-II' and HDL cholesterol (40) in addition to increased cholesterol transfer from VLDL to HDL upon increased triglyceride hydrolysis (41).

Our analyses also show that use of lipid-lowering therapy, which was predominantly statins, was associated with significantly higher apoC-II' truncation. Further studies are needed to identify whether this reflects a direct effect of statin medication and whether this contributes to some of the known clinical benefits of statin therapy. From a clinical perspective, the finding of reduced apoC-I truncation with DPP-4 inhibitors may indicate an off-target effect that may counteract some of their cardiometabolic benefits.

A main strength of the study is that we were able to perform these posttranslational measures of apoC-I and apoC-II in a large demographically diverse longitudinal cohort with a standardized approach to clinical and laboratory data collection. In the majority of subjects, we were able to combine proteoform distribution with total plasma concentrations measured by enzymatic assay. This not only allowed us to compare relationships of proteoform distribution with total values more directly but also allowed calculation of individual truncated and native molecule concentration. Importantly, the large cohort size allowed robust statistical modeling with adjustment for many relevant covariates.

Although the cohort size and comprehensive phenotyping permitted robust statistical modeling, the study conclusions are based on association analyses. Many of the novel relationships need to be examined by more direct mechanistic models to confirm causality and identify underlying mechanisms. Since both apoC-I and apoC-II are interchangeable proteins between different lipoprotein particles, measurements in whole plasma cannot distinguish the particle-specific effects. Because of funding limitations, total apoC-I and apoC-II concentrations at baseline were measured only in individuals who had also available exam 5 samples. Similar association patterns of apoC-I and apoC-II proteoform distribution with clinical characteristics and plasma lipids between those with and without total concentration measurements indicate a very limited selection bias.

In conclusion, our study highlights posttranslational modification of apoC-I and apoC-II as an important component of short-term and long-term regulation of plasma lipids. Enhanced understanding of the mechanisms of posttranslational regulation of apoC-I and apoC-II may provide insights into the pathophysiology of disturbances in lipid metabolism as well as identify new therapeutic targets. Finally, as other apolipoproteins, such as apoC-III, have additional lipid-independent proatherogenic effects (42), examination of similar possibilities for apoC-I and apoC-II seems warranted.

## Data availability

The data and study materials will be made available to other researchers for the purposes of reproducing the results only with proper institutional review board approvals and strict adherence to cohort-specific regulations. Requests for data can be directed to J. K. or P. D. R.

## Supplemental data

This article contains supplemental data.

## Conflict of interest

The contents of this article do not represent the views of the Department of Veterans Affairs or the United States Government. J. F. is currently an employee of Biogen. All other authors declare that they have no conflicts of interest with the contents of this article.
