# Supplementary material for: Plasma proteoforms of apolipoproteins C-I and C-II are associated with plasma lipids in the Multi-Ethnic Study of Atherosclerosis
Source: J Lipid Res. 2022 Aug 9;63(9):100263. doi: 10.1016/j.jlr.2022.100263 (PMC9494236; doi:10.1016/j.jlr.2022.100263)
Supplement: Supplemental Methods and Tables S1–S3 [file mmc1.docx]

**Online Supplement**

*Mass spectrometry immunoassay*

Polyclonal goat anti-human antibodies to apoC-I (Cat. No. 31A-G1b) and apoC-II (Cat. No. 32A-G2b) were obtained from Academy Biomedical (Houston, TX). Acetone (UN1090) was from JT Baker (Radnor, PA). Hydrochloric acid (HCl; AB06037), trifluoroacetic acid (TFA, AB02010), and acetonitrile (ACN; AB00120) were from AmericanBio (Natick, MA). N-methylpyrrolidinone (NMP; BP1172-4), 1,1ʹ carbonyldiimidazole (97%) (CDI, 115533), phosphate buffered saline buffer (PBS, 28372), 2-(N-morpholino) ethanesulfonic acid (MES) saline buffer (28390), and Mass Spectrometric Immunoassay (MSIA) Tips (991CUS01) were acquired from Thermo Fisher Scientific (Waltham, MA). Tween20 (Cat. No. P7949), sinapic acid (85429), and ethanolamine (ETA; 398136) were obtained from Sigma Aldrich (St. Louis, MO).

Activation and derivatization of the microcolumns inside the MSIA Tips were performed on a Multimek 96 automated 96-channel pipettor (Beckman Coulter, Brea, CA). The MSIA Tips were first rinsed with 200 mM HCl (10 aspiration/dispense cycles, 100 μL each), followed by water (10 cycles) and acetone (10 cycles). Then, the microcolumns inside the tips were activated with CDI (100 mg/mL in NMP (1,000 cycles, 50 μL each) followed by two rinses with NMP (10 cycles each, 100 μL). The activated tips were immersed into wells containing 0.32 μg apoC-I antibody and 2.25 μg apoC-II antibody/well (in 100 μL of 10 mM MES buffer), and 1000 cycles (50 μL each) were performed, allowing for antibody attachment to the activated microcolumns. The tips were then rinsed with ETA and two rinses with PBS (50 cycles each, 100 μL). The total time taken for activation and derivatization of 96 MSIA Tips was 1.5 h. The antibody-derivatized tips were stored at 4°C until use.

Plasma samples were thawed and diluted immediately prior to running the assays. The first dilution (S1) was prepared by mixing 3 μL plasma with 117 μL of PBS, 0.1% Tween (PBST). Then, 40 μL of the S1 dilution was mixed with 120 μL of PBST, yielding 160 μL analytical plasma samples (S2 dilution).

The antibody-derivatized tips were mounted onto the head of the Multimek 96 pipettor and first rinsed with PBST (10 cycles, 100 μL). The tips were then immersed into the wells of a microplate containing the analytical samples, and 250 cycles (100 μL each) were performed, allowing for affinity capture of the targeted proteins. Then, one rinse with PBST (100 cycles, 100 μL) and two rinses with water (10 cycles each, 100 μL) followed to wash off the nonspecifically bound proteins from the microcolumns. To elute the captured proteins, 5 μL of MALDI matrix (20 g/L sinapic acid in 33% (v/v) ACN and 0.4% (v/v) TFA) was aspirated into each tip, pushed up and down three times, and then dispensed directly onto a 96-well formatted MALDI target. Sample spots were dried on a hot plate at 50°C. Samples were run in batches of 96; each batch contained 90 analytical samples and 6 quality control samples (two distinct plasma samples aliquoted in triplicate), which were used to calculate intra- and inter-assay coefficients of variations.

Bruker Autoflex III MALDI-TOF instrument (Bruker, Billerica, MA) was utilized to acquire linear mass spectra. The instrument was operated in positive ion mode with 20.00 kV and 18.45 kV ion source voltages. The mass spectra were acquired in the mass range from 5 to 20 kDa, with 50 ns delay, and signal suppression of up to 4,500 Da. A total of 1,000 laser-shots were acquired and summed for each mass spectrum. Singly charged peaks were observed for truncated apoC-I’ (m/z=6428.4), native C-I (m/z=6630.6), truncated apoC-II’ (m/z=8204.2), and native apoC-II (m/z=8914.9).

The mass spectra were first externally calibrated with protein calibration standards and then internally calibrated using the highest intensity apoC(-s) signals. The spectra were baseline subtracted (Convex Hull algorithm, 0.8 flatness) and smoothed (Savizky Golay algorithm, 5 m/z width and 1 cycle) using Flex Analysis software (Bruker Daltonics). Areas under the peaks for native and truncated proteoforms of apoC-I and apoC-II signals were integrated using Zebra 1.0 software (Intrinsic Bioprobes Inc., Tempe, AZ). To obtain the percent abundance of truncated apoC-I, the peak area of truncated apoC-I was divided with the sum of peak areas of both truncated and full-length apoC-I. The percent abundance of truncated apoC-II was similarly calculated.

*Enzyme-immunoassay for total apoC-I and apoC-II*

Plasma concentrations of total apoC-I and apoC-II were determined in 3,851 participants who had available samples from both exams using sandwich ELISAs with identical detection antibodies as used in the proteoform assays above. Plasma samples were diluted 1:40,000 in Tween-containing diluent (1× PBS/2% BSA/0.05% Tween 20) and loaded in duplicate onto 96-well plates coated with apoC-I or apoC-II antibodies [0.5 μg/well]. Plates were incubated for 1 h at 37°C and washed three times with 1× PBS with 0.1% Tween 20. Detection antibodies conjugated to horseradish peroxidase or biotin were added [0.1 μg/well] (Academy Bio-Medical Co.) and plates were incubated for 1 h at 37°C followed by three washes with 1× PBS with 0.1% Tween 20. For all plates, color was developed by incubation with o-phenylenediamine solution (Sigma-Aldrich, St. Louis, MO), and absorbance of each well was determined at 450 nm using a 96-well plate reader. Each plate contained a calibration curve and two control samples to allow quality-control assessments. Replicates with a coefficient of variation >15% were repeated.

**Table S1:** Demographic and clinical characteristics of the cohort at baseline and follow-up. Data are means ± SD or percentages.

| **Variable** | **Baseline**  **(n=5,791)** | **Follow-up**  **(n=3,851)** |
| --- | --- | --- |
| **Age (Years)** | 62 ± 10 | 70 ± 9 |
| **Race ethnicity** |  |  |
| **Non-Hispanic White (%)** | 37% | 39% |
| **African American (%)** | 29% | 27% |
| **Hispanic American (%)** | 22% | 21% |
| **Chinese American (%)** | 12% | 12% |
| **Gender (% Women)** | 52% | 52% |
| **BMI (kg/m^2^)** | 28.3 ± 5.5 | 28.4 ± 5.6 |
| **Hypertension (%)** | 46% | 60% |
| **Fasting glucose (mg/dl)** | 97 ± 30 | 102 ± 29 |
| **Impaired fasting glucose (%)** | 14%% | 21% |
| **Diabetes (%)** | 13% | 20% |
| **Triglycerides (mg/dl)** | 131 ± 88 | 108 ± 60 |
| **Total cholesterol (mg/dl)** | 194 ± 35 | 182 ± 37 |
| **LDL-cholesterol (mg/dl)** | 117 ± 31 | 105 ± 32 |
| **HDL-cholesterol (mg/dl)** | 50 ± 14 | 56 ± 17 |
| **Lipid-lowering therapy** (%) | 17% | 39% |
| **Statins** | 91% | 96% |
| **eGFR (ml/min/1.73 m^2^)** | 89 ± 21 | 79 ± 21 |

**Table S2:** Spearman correlations between total plasma concentration and truncated- to-native proteoform ratios of apoC-I and apoC-II at baseline and follow-up.

|  |  | **Total apoC-I** | | **Total apoC-II** | | **apoC-I'/C-I** | |
| --- | --- | --- | --- | --- | --- | --- | --- |
|  | *Exam:* | *Baseline* | *Follow-up* | *Baseline* | *Follow-up* | *Baseline* | *Follow-up* |
| **Total apoC-II** | rho | 0.57 | 0.57 | - | - | - | - |
|  | P-value | <0.0001 | <0.0001 | - | - | - | - |
| **apoC-I'/C-I** | rho | 0.11 | 0.20 | 0.02 | 0.10 | - | - |
|  | P-value | <0.0001 | <.0001 | 0.3 | <0.0001 | - | - |
| **apoC-II'/C-II** | rho | 0.26 | 0.41 | 0.20 | 0.27 | 0.36 | 0.41 |
|  | P-value | <.0001 | <.0001 | <.0001 | <.0001 | <0.0001 | <0.0001 |

**Table S3.** Multivariable association of truncated-to-native proteoform ratios of apoC-I and apoC-II with demographic and cardiometabolic characteristics at baseline in participants with (n=3,851) and without (n=1,940) total apoC-I and apoC-II concentrations measurement. Data are β-estimates (SE). *^a^*p<0.05 exposure effect, *^b^*p<0.05, interaction effect with availability of total concentrations. Continuous variables were standardized to 1 SD of natural log-transformed values, categorical variables are compared with the reference group. NHW, non-Hispanic whites. eGFR, estimated glomerular filtration rate

|  | **ApoC-I’/C-I** | | **ApoC-II’/C-II** | |
| --- | --- | --- | --- | --- |
| **Variable** | **Total** | **No total** | **Total** | **No total** |
| **Age (1 SD)** | -0.02 (0.02) | 0.01 (0.02) | -0.02 (0.02) | -0.08 (0.02)*^a,b^* |
| **Gender (Women)** | -0.03 (0.03) | -0.03 (0.04) | 0.35 (0.03)*^a^* | 0.27 (0.03)*^a^* |
| **Race/ethnicity (vs. NHW)** |  |  |  |  |
| **African American** | 0.27 (0.04)*^a^* | 0.15 (0.06)*^a^* | 0.59 (0.04)*^a^* | 0.60 (0.06)*^a^* |
| **Chinese American** | -0.37 (0.05)*^a^* | -0.34 (0.07)*^a^* | -0.04 (0.05) | -0.04 (0.07) |
| **Hispanic** | 0.11 (0.04)*^a^* | 0.12 (0.06)*^a^* | 0.07 (0.04) | 0.05 (0.06) |
| **Body-mass index (1 SD)** | -0.23 (0.02)*^a^* | -0.18 (0.02)*^a^* | -0.12 (0.02)*^a^* | -0.08 (0.02)*^a^* |
| **Fasting glucose (1 SD)** | -0.13 (0.02)*^a^* | -0.14 (0.02)*^a^* | -0.02 (0.02) | -0.02 (0.02) |
| **Lipid-lowering therapy** | -0.02 (0.04) | 0.05 (0.06) | 0.15 (0.04)*^a^* | 0.20 (0.06)*^a^* |
| **eGFR (1SD)** | -0.15 (0.02)*^a^* | -0.13 (0.02)*^a^* | -0.12 (0.02)*^a^* | -0.07 (0.02)*^a^* |
| **Lipids (adj. for above):** |  |  |  |  |
| **Triglycerides (1 SD)** | -0.28 (0.01)*^a^* | -0.25 (0.02)*^a^* | -0.13 (0.02)*^a^* | -0.08 (0.02)*^a,b^* |
| **HDL cholesterol (1 SD)** | 0.03 (0.01) | 0.03 (0.02) | 0.36 (0.01)*^a^* | 0.33 (0.02)*^a^* |
